# Toll-Like Receptor 2 Mediates Ischemia-Reperfusion Injury of the Small Intestine in Adult Mice

**DOI:** 10.1371/journal.pone.0110441

**Published:** 2014-10-16

**Authors:** Toshio Watanabe, Tetsuya Tanigawa, Atsushi Kobata, Shogo Takeda, Yuji Nadatani, Koji Otani, Hirokazu Yamagami, Masatsugu Shiba, Kazunari Tominaga, Yasuhiro Fujiwara, Tetsuo Arakawa

**Affiliations:** 1 Department of Gastroenterology, Osaka City University Graduate School of Medicine, Osaka, Japan; 2 Department of Pharmacology and Experimental Therapeutics, Kyoto Pharmaceutical University, Kyoto, Japan; University of Missouri, United States of America

## Abstract

Toll-like receptor 2 (TLR2) recognizes conserved molecular patterns associated with both gram-negative and gram-positive bacteria, and detects some endogenous ligands. Previous studies demonstrated that in ischemia-reperfusion (I/R) injury of the small intestine, the TLR2-dependent signaling exerted preventive effects on the damage in young mice, but did not have a significant effect in neonatal mice. We investigated the role of TLR2 in adult ischemia-reperfusion injury in the small intestine. Wild-type and TLR2 knockout mice at 16 weeks of age were subjected to intestinal I/R injury. Some wild-type mice received anti-Ly-6G antibodies to deplete circulating neutrophils. In wild-type mice, I/R induced severe small intestinal injury characterized by infiltration by inflammatory cells, disruption of the mucosal epithelium, and mucosal bleeding. Compared to wild-type mice, TLR2 knockout mice exhibited less severe mucosal injury induced by I/R, with a 35%, 33%, and 43% reduction in histological grading score and luminal concentration of hemoglobin, and the numbers of apoptotic epithelial cells, respectively. The I/R increased the activity of myeloperoxidase (MPO), a marker of neutrophil infiltration, and the levels of mRNA expression of tumor necrosis factor-α (TNF-α), intercellular adhesion molecule-1 (ICAM-1), and cyclooxygenase-2 (COX-2) in the small intestine of the wild-type mice by 3.3-, 3.2-, and 13.0-fold, respectively. TLR2 deficiency significantly inhibited the I/R-induced increase in MPO activity and the expression of mRNAs for TNF-α and ICAM-1, but did not affect the expression of COX-2 mRNA. I/R also enhanced TLR2 mRNA expression by 2.9-fold. TLR2 proteins were found to be expressed in the epithelial cells, inflammatory cells, and endothelial cells. Neutrophil depletion prevented intestinal I/R injury in wild-type mice. These findings suggest that TLR2 may mediate I/R injury of the small intestine in adult mice via induction of inflammatory mediators such as TNF-α and ICAM-1.

## Introduction

Ischemia-reperfusion (I/R) injury of the small intestine occurs in a variety of clinical conditions, including small bowel occlusion and thrombosis of mesenteric artery, vascular surgery, shock, small bowel transplantation, and trauma [1]. Reperfusion after ischemia in the small intestine initiates inflammatory responses, resulting in cell and tissue injuries. Subsequently, intestinal barrier dysfunction causes the translocation of bacteria and noxious substances into the circulation [2]. Gastrointestinal I/R also causes intestinal dysmotility, which is associated with the disruption of the interstitial cells of Cajal network [3] and changes in synthesis of several endogenous mediators such as ghrelin [4], which induces bacterial overgrowth and bacterial translocation from the gastrointestinal tract. Consequently, these phenomena lead to systemic inflammation and produce the multiple organ dysfunction syndrome. Thus, I/R injury of the small intestine is a clinically important problem associated with high mortality and morbidity. Although several inflammatory pathways are postulated to modulate inflammation during intestinal I/R injury [5], [6], [7], [8], the precise mechanisms of I/R-induced inflammations remain unclear.

Toll-like receptors (TLRs) are pattern-recognizing receptors that can be activated by microbial components or endogenous host molecules [9]. So far, 10 and 12 functional TLRs have been identified in humans and mice, respectively, with TLR1–TLR9 being conserved in both species. Ligands binding to TLRs initiate signals that involve many kinases, including nuclear factor-κB and mitogen-activated protein kinases, resulting in the induction of numerous cytokines [10]. These TLRs have been reported to play crucial roles in a variety of gastrointestinal diseases [11], [12], [13], and recent studies suggest that TLRs participate in inflammatory signaling pathways during I/R injury in several organs including the small intestine [14], [15].

TLR2, a member of the TLR family, recognizes conserved molecular patterns associated with both gram-negative and gram-positive bacteria. These molecules include lipopeptides/lipoproteins, lipoteichoic acid, zymosan, and components of peptidoglycan [16]. TLR2 also detects some endogenous ligands, including heat shock proteins, saturated fatty acids and high-mobility group box proteins 1 [17] that play a crucial role in I/R injuries in several organs [14]. It is important to note, however, that evidence regarding the roles of TLR2 in intestinal I/R is inconclusive: TLR2 deficiency have worse I/R-induced intestinal injury in young mice (4 weeks old) [18], while no difference in the I/R injury was found in neonatal mice (2 weeks old) between TLR 2 knockout (KO) and wild-type mice [19]. Thus, the roles of TLRs in the I/R injury of the small intestine may differ at different ages. In this study, we investigated the role of TLR2 in the intestinal I/R injury in adult mice.

## Materials and Methods

### Animals and induction of I/R injury of the small intestine

TLR2 KO mice that were backcrossed 8 times on the C57BL/6 background, were obtained from Oriental Bioservice Inc (Kyoto, Japan). Wild-type mice (C57BL/6) were purchased from Charles River Japan Inc (Atsugi, Japan) and used as the control strain for TLR2 KO mice. All animals were housed in polycarbonate cages with paper-chip bedding in an air-conditioned biohazard room with a 12-h light-dark cycle. All animals had free access to food and water. All experiments were performed in mice at 16 weeks of age.

To induce injury, the superior mesenteric artery was clamped (ischemia) under urethane anesthesia (1.25 g/kg i.p.), and reperfusion was established 45 min later by the removal of the clamp. The intestine was excised after a 60 min reperfusion period, and intestinal injury after reperfusion was evaluated. To investigate the role of neutrophils, some animals were intraperitoneally administered either rat anti-mouse anti-Ly-6G (clone: 1A8) or control rat IgG2a (both from BD Pharmingen, San Diego, CA) at a dose of 300 µg 24 h before I/R treatment. All experiments were performed with at least 5 mice per treatment group. All experimental procedures were approved by the Animal Care Committee of the Osaka City University Graduate School of Medicine (Permit Number: 09015). All surgery was performed under urethane anesthesia, and all efforts were made to minimize suffering

### Assessment of intestinal mucosal injury induced by I/R

Intestinal injury after reperfusion was evaluated by a measurement of the luminal concentration of hemoglobin (a marker of intestinal bleeding), histological examination, and counting of apoptotic cells. After reperfusion, the animals were killed by CO2 inhalation. The small intestines were removed, and cut 20 cm above the terminal ileum. After making a 20-cm intestinal loop, saline (1 mL) was injected into the loop. The luminal contents were aliquoted after centrifugation. Intestinal bleeding was quantified indirectly as hemoglobin concentration in the luminal lavage fluid using a kit (Wako Pure Chemical Industries, Ltd., Osaka, Japan) and following the manufacturer's protocol.

A sample tissue, 4.5 cm in length, was removed from the middle portion of the 20 cm long small intestine and cut into three pieces of equal length (1.5 cm each). The proximal, middle, and distal samples were subjected to a myeloperoxidase (MPO) activity assay, histological examination, and assays to determine the mRNA levels and the prostaglandin (PG) E_2_ concentration, respectively. For the histological evaluation, segments of the ileum were fixed in 0.1 M phosphate buffer (pH 7.4) containing 4% paraformaldehyde and embedded in OCT compound (Miles, Elkhart, IN). Then, the cryostat sections were stained with hematoxylin-eosin and histopathological examination of the I/R tissue was performed by employing a staging method described by Hierholzer et al [20], in which the severity of the injury was graded from 0 to 4. The different grades were defined as follows: Grade 0, no specific pathological changes are observed (i.e., normal architecture of the gut wall, including villi, crypts, lamina propria, and muscularis externa); Grade 1, mild mucosal damage is observed (i.e., denudation of villi epithelium, but otherwise normal structure); Grade 2, moderate damage has occurred (i.e., loss of villus length and epithelial sloughing with evidence of congestion, hemorrhage, and inflammation in the mucosa, but no change in the submucosa or muscularis externa); Grade 3, extensive damage is observed (i.e., loss of a large number of villi, including denudation, sloughing, and the presence of granulomatous tissue; the damage is localized to submucosa and muscularis); Grade 4, there is severe damage and necrosis (i.e., inflammation and necrosis in areas throughout the thickness of the intestinal wall).

We also evaluated the intestinal I/R injury by counting apoptotic epithelial cells using immunohistochemistry with an antibody against single-stranded DNA (ssDNA). The immunohistochemical methods are described below. The apoptotic index was defined as the percentage of labeled epithelial cells in a total count of>500 epithelial cells.

### Determination of mRNA levels by real-time quantitative reverse transcription-polymerase chain reaction

Total RNA was extracted from the small intestinal tissue using an ISOGEN kit (Nippon Gene Co., Ltd. Tokyo, Japan). Real-time quantitative reverse transcription-polymerase chain reaction (RT-PCR) analyses were performed as previously described [21]. Expression of mRNAs for TLR2, tumor necrosis factor (TNF)-α, intercellular adhesion molecule (ICAM)-1, and cyclooxygenase (COX)-2 was normalized for the expression of glyceraldehyde-3-phosphate dehydrogenase mRNA and expressed as ratio to the mean value for small intestinal tissue of sham-operated mice. The sequences of PCR primers and TaqMan probes are as follows: For the mouse TLR2, the sense primer was 5′-CTCTGGAGCATCCGAATTGC-3′, the anti-sense primer was 5′-GCTGAAGAGGACTGTTATGGC-3′, and the TaqMan probe was 5′-CCTCAGACAAAGCGTCAAATCTCAGAGGA- 3′. For mouse TNF-α, the sense primer was 5′-TCATGCACCACCATCAAGGA-3′, the anti-sense primer was 5′-GAGGCAACCTGACCACTCTCC-3′, and the TaqMan probe was 5'-AATGGGCTTTCCGAATTCACTGGAGC-3'. For mouse ICAM-1, the sense primer was 5′-GGCTGAAAGATGAGCTCGAGAG-3′, the anti-sense primer was 5′-TTCTCAAAGCACAGCGGACTG -3′, and the TaqMan probe was 5′-GAAGCTGTTTGAGCTGAGCGAGATCGG -3′. For mouse COX-2, the sense primer was 5'-AAGCCCTCTACAGTGACATCGA-3', the antisense primer was 5'-ATGGTCTCCCCAAAGATAGCAT-3', and the TaqMan probe was 5'-CTGCTGGTGGAAAAACCTCGTCCA-3'. Real-time quantitative RT-PCR analyses were performed using an Applied Biosystems ABI 7500 Fast RT-PCR system and software (Life Technologies, Foster City, CA).

### Immunohistochemical study

Cryostat sections, cut serially at 6 µm thickness, were mounted on silanized slides (Dako Japan, Tokyo, Japan), and a rabbit polyclonal antibody to ssDNA (Immuno-Biological Laboratories Co., Ltd., Fujioka, Japan) or TLR2 (Santa Cruz Biotechnology, Inc., Santa Cruz, CA). Immunohistochemical staining was performed with a streptavidin-biotin peroxidase method according to the manufacturer's instructions with the ImmunoCruz rabbit LSAB Staining System (Santa Cruz Biotechnology, Inc.). Counterstaining was performed with methyl green.

### Measurement of MPO activity

MPO activity in small intestinal tissue, a marker of neutrophil infiltration, was assayed as previously described [22]. One unit of MPO activity was defined as that which degrades 1 µmol of peroxide per min at 25°C. Proteins were measured with a modified bicinchoninic acid method. Results are expressed in units per gram of tissue protein.

### Assay of the PGE_2_ concentration in small intestinal tissue

Small intestinal tissue was weighed, and placed in a tube containing 100% methanol and 0.1 mM indomethacin (Wako Pure Chemical Industries, Ltd.) Then, the tissues were homogenized by using a Polytron homogenizer (IKA, Tokyo, Japan) and centrifuged at 12,000 rpm for 10 min at 4°C. After the supernatant of each sample had been evaporated with N_2_ gas, the residue was resolved in assay buffer and used for determination of PGE_2_. The concentrations of PGE_2_ were measured by using an enzyme immunoassay (PGE_2_ EIA kit, Cayman Chemical, Ann Arbor, MI).

### Statistical Analysis

Values are presented as means ± SD. One-way analysis of variance was used to test for significance of differences among treatment group means, and results were examined by Fisher's protected least-significant-difference test. Differences with *P* values less than 0.05 were considered significant.

## Results

### Effect of TLR2 deficiency on the severity of I/R injury of the small intestine

**Figure 1:**
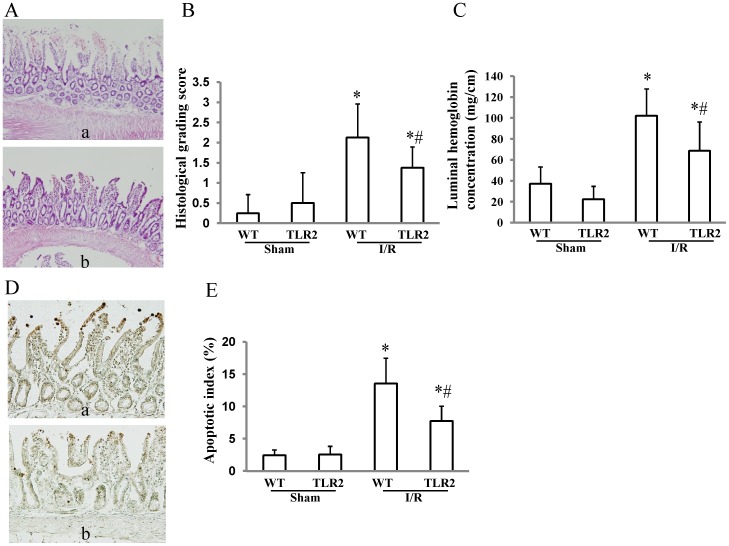
Effect of TLR2 deficiency on intestinal ischemia/reperfusion injury. Wild-type (WT) mice and TLR2 knockout (KO) mice were subjected to 45 min of ischemia followed by 60 min of reperfusion. After removal of the small intestine, intestinal injuries were assessed by using the histological grading system, measuring the luminal hemoglobin concentration, and counting apoptotic epithelial cells with an antibody against ssDNA. (A) Histological findings of intestinal ischemia/reperfusion (I/R) injury in WT (a) and TLR2 KO (b) mice. Compared to WT mice (a), TLR2 KO mice exhibited less severe intestinal injury induced by 45 min of ischemia followed by 60 min of reperfusion (b). (B) Comparison of the histology grading scores between WT and TLR2 KO mice that were subjected to I/R injury. Each column represents the mean ± SD. N = 8. *P<0.01 vs WT mice with sham operation, #*P*<0.05 vs WT mice subjected to I/R injury. (C) Comparison of luminal hemoglobin concentrations between WT and TLR2 KO mice that were subjected to I/R injury. Each column represents the mean ± SD. N = 6–8. *P<0.01 vs WT mice with sham operation, #*P*<0.01 vs WT mice subjected to I/R injury. (D) Immunohistochemical staining of ssDNA in intestinal mucosa. I/R resulted in induction of apoptosis of epithelial cells labeled with an antibody against the ssDNA of injured mucosa in WT mice (a), and TLR2 deficiency prevented I/R-induced apoptosis of the small intestinal epithelial cells (b). (E) Comparison of apoptotic indices between WT and TLR2 KO mice that were subjected to I/R injury. Each column represents the mean ± SD. N = 8. *P<0.01 vs WT mice with sham operation, #*P*<0.01 vs WT mice subjected to I/R injury.

In wild-type mice, I/R induced severe small intestinal injury characterized by infiltration by inflammatory cells, disruption of the mucosal epithelium, and mucosal bleeding (Figure 1A). Compared to wild-type mice, TLR2 KO mice exhibited less severe mucosal injury induced by I/R, with 35% and a 33% reduction in the histological grading score (Figure 1B) and luminal concentration of hemoglobin (Figure 1C), respectively. Furthermore, TLR2 deficiency prevented I/R-induced apoptosis of the small intestinal epithelial cells (Figure 1D): The apoptotic indices of TLR2 KO mice were reduced by 43% compared with those of wild-type mice (Figure 1E).

### Effect of TLR2 deficiency on expression of inflammatory mediators, neutrophil infiltration, and PGE_2_ synthesis during development of the intestinal I/R injury

**Figure 2:**
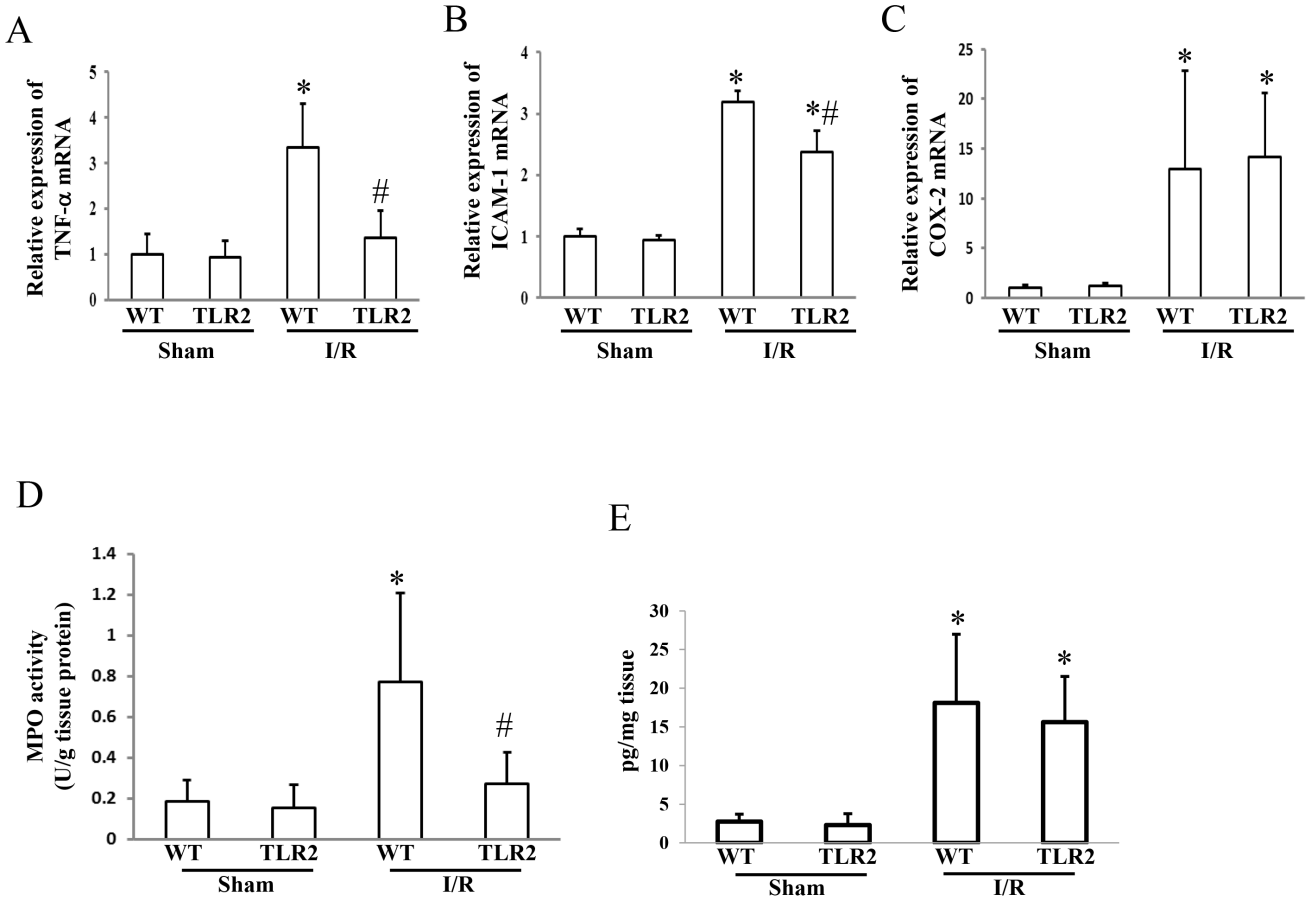
Effect of TLR2 deficiency on the expression of inflammatory mediators, neutrophil infiltration, and prostaglandin E_2_ synthesis after the ischemia-reperfusion injury of the small intestine. Wild-type (WT) mice and TLR2 knockout mice were subjected to 45 min of ischemia, followed by 60 min of reperfusion. After removing the small intestine, the mRNA expression levels for the tumor necrosis factor (TNF)-α (A), the intercellular adhesion molecule (ICAM)-1 (B), and cyclooxygenase (COX)-2 (C) were determined. Assays were also performed to estimate the myeloperoxidase (MPO) activity (a marker of neutrophil infiltration, D), and the prostaglandin E_2_ concentrations (E). Each column represents the mean ± SD. N = 6. *P<0.01 vs WT mice with sham operation, #*P*<0.01 vs WT mice subjected to the ischemia-reperfusion injury. I/R; ischemia/reperfusion

The intestinal I/R treatment increased expression levels of mRNAs for TNF-α, ICAM-1, and COX-2 in the small intestine of wild-type mice by 3.3-, 3.2-, and 13.0-fold, respectively (Figures 2A-2C). TLR2 deficiency prevented the I/R-induced increase in mRNAs for TNF-α and ICAM-1, but did not affect that in COX-2 mRNA. The I/R treatment also increased MPO activities in wild-type mice by 4.1-fold, but this effect was markedly inhibited in TLR2 KO mice (Figure 2D).

The I/R increased the PGE_2_ concentration in the small intestine of the wild-type as well as the TLR2 KO mice. The TLR2 deficiency did not affect the I/R-induced increase in the synthesis of PGE_2_ (Figure 2E).

### Roles of neutrophils in intestinal I/R injury

**Figure 3:**
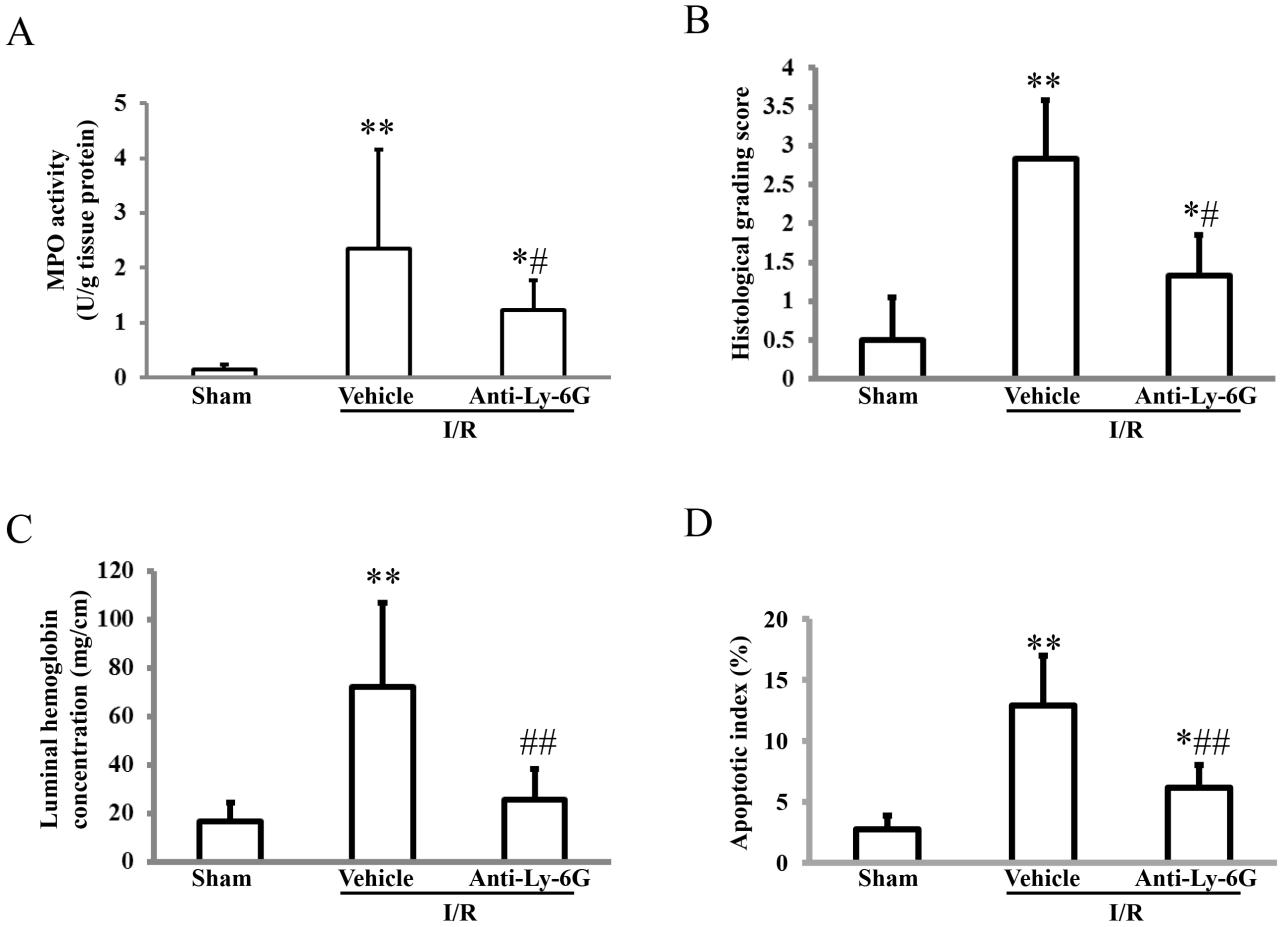
Roles of neutrophils in the intestinal ischemia-reperfusion injury. Wild-type mice that were intraperitoneally administered either rat anti-mouse Ly-6G antibodies or the vehicle (control rat IgG2a) 24 h before were subjected to 45 min of ischemia followed by 60 min of reperfusion. After removal of the small intestine, an assay of myeloperoxidase (MPO) activity (a marker of neutrophil infiltration, A), assessment of the intestinal injury by using the histological grading system (B), measuring the luminal hemoglobin concentration (C), and counting apoptotic epithelial cells with an antibody to ssDNA (D) were each performed. Each column represents the mean ± SD. N = 6–9. **P<0.01, * *P*<0.05 vs mice with sham operation, ##*P*<0.01, #*P*<0.05 vs mice that were subjected to the ischemia-reperfusion injury and given the vehicle. I/R; ischemia/reperfusion.

Neutrophil depletion by rat anti-mouse anti-Ly-6G antibodies inhibited the I/R-induced increase in MPO activity by 47% (Figure 3A), and resulted in prevention of the intestinal I/R injury. The histological grading score (Figure 3B), luminal concentration of hemoglobin (Figure 3C), and apoptotic indices (Figure 3D) in mice treated anti-Ly-6G antibodies were reduced by 53%, 64%, and 52%, respectively.

### Expression of TLR2 during development of intestinal I/R injury

**Figure 4:**
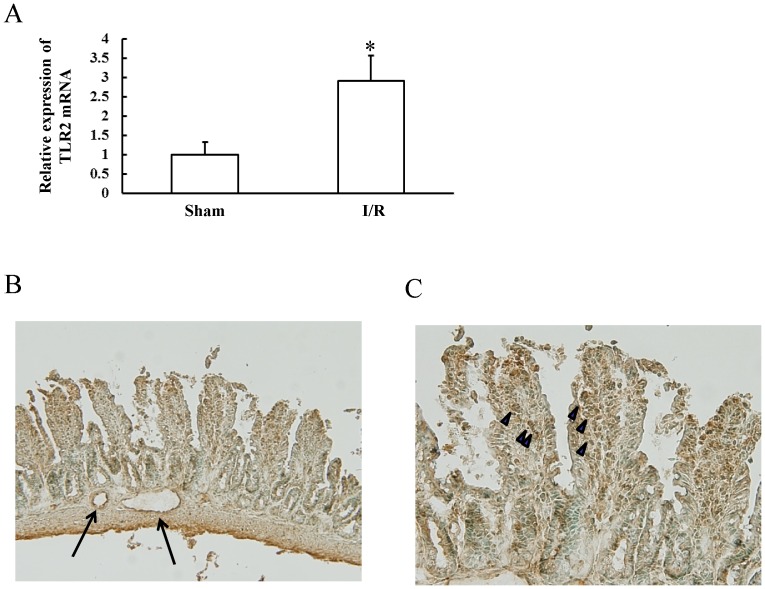
Expression of TLR2 during the development of the intestinal ischemia-reperfusion injury. Wild-type mice were subjected to 45 min of ischemia followed by 60 min of reperfusion. After removal of the small intestine, expression of TLR2 was examined by real-time RT-PCR and immunohistochemical staining. (A) Effect of the ischemia/reperfusion (I/R) treatment on intestinal TLR2 mRNA. Each column represents the mean ± SD. N = 6. *P<0.01 vs mice with sham operation. (B, C) Immunohistochemical staining for TLR2. TLR2 proteins were expressed in epithelial cells, inflammatory cells (arrow heads), and endothelial cells (arrows) in the injured small intestine.

The I/R treatment enhanced TLR2 mRNA expression by 2.9-fold (Figure 4A). Upon immunohistochemical examination, TLR2 proteins were found to be expressed in epithelial cells, inflammatory cells, and endothelial cells in the injured small intestine (Figures 4B and 4C).

## Discussion

In contrast to the results of previous studies [18], [19], our results indicate that adult TLR2 KO mice exhibited less severe I/R injury than neonatal mice, as indicated by 3 different assessments (a histological grading system, luminal hemoglobin concentration, and an apoptotic index). The results suggest that the TLR2-dependent pathway mediates I/R injury in adult mice, and that the roles of TLR2 in intestinal I/R injury may differ with age. Similar to TLR2, an age-dependent role of TLR4 in intestinal I/R injury has also been reported; TLR4 was protective against intestinal I/R injury in neonatal mice (2 weeks old) [19], and it mediated the damage in adult mice (8–14 weeks old) [23]. In addition to inflammatory signals that lead to tissue injury, TLR2 induces protective signals that result in production of cytoprotective molecules such as heat shock proteins [24], tight junction proteins [25], [26], heme oxygenase-1 [27], and Bcl2 (an anti-apoptotic molecule) [24]. Therefore, the overall role of TLR2 in intestinal I/R injury may depend on the balance between inflammatory and protective signals. Changes in gut bacterial flora [28] and TLR2 expression levels [29] from birth to adulthood may affect such a balance. Since genetic depletion of TLR2 prevented intestinal I/R injury, TLR2-dependent inflammatory signals seem to be stronger than TLR2-dependent protective signals during development of the injury in adult mice.

Adhesion molecules such as ICAM-1 mediate leukocyte infiltration into inflamed tissue, and are involved in many types of intestinal injuries [30], [31] In this study, we demonstrated that genetic depletion of TLR2 ameliorated the I/R-induced infiltration of neutrophils into the small intestine by inhibiting the overexpression of ICAM-1. The depletion of circulating neutrophil not only prevented the increase of MPO activity in the small intestine, but also attenuated the I/R injury. Hence, we believe that this inhibition of ICAM-1 expression may be responsible for the resistance to such injury in TLR-2 KO mice. The finding that neutralizing antibodies against ICAM-1 prevented the intestinal I/R injury with a decrease in neutrophil infiltration in rats [32] supports our notion.

ICAM-1 expression is up-regulated in response to a variety of inflammatory mediators, including pro-inflammatory cytokines, hormones, and cellular stresses [33]. Among these mediators, TNF-α is a strong inducer of ICAM-1 expression [34], leading to tissue injuries including gastrointestinal ulceration [35]. Therefore, prevention of overexpression of TNF-α may contribute to the inhibition of ICAM-1 expression in TLR2 KO mice. Our immunohistochemical analysis demonstrated abundant expression of TLR2 on the endothelial cells of the small intestine. A recent study reported that activation of endothelial TLR2 directly induced expression of ICAM-1 as well as inflammatory cytokines in human umbilical vein endothelial cells [36], suggesting that TLR2 and inflammatory cytokines such as TNF-α may act in concert to induce ICAM-1 expression during the development of intestinal I/R injury.

In a previous study, we demonstrated that activation of the signaling pathway of MyD88, an adaptor molecule of all TLRs, except TLR3, inhibited I/R injury in the small intestine via induction of COX-2 expression and PGE_2_ synthesis [37]. Although some reports indicated involvement of the TLR2/MyD88-dependent signaling pathway in the induction of COX-2 [38], [39], [40], we found that TLR2 deficiency did not affect COX-2 expression and PGE_2_ synthesis in the small intestine during the development of intestinal I/R injury. Several lines of evidence demonstrated that the TLR4/MyD88-dependent pathway induced COX-2 expression, leading to prevention of damage and maintenance of mucosal integrity in the gastrointestinal tract [41], [42], [43]. Therefore, TLR4 might mediate COX-2 expression in intestinal I/R injury.

In this study, we used urethane for anesthesia. Urethane is widely used as an anesthetic for animal studies because urethane minimally affects the physiological conditions and responses. However, urethane has been reported to affect some of the pathophysiological conditions in rats. Under physiological conditions, this anesthetic drug alters the blood levels of glucose, epinephrine, and several endocrine hormones [44], [45]. It also suppresses the expression of inflammatory mediators, such as, COX-1, COX-2, and the inducible nitric oxide synthase, during rat lung inflammation [46]. Additionally, the intraperitoneal injection of urethane damages the intra-abdominal organs [44]. However, in a preliminary study, we found that the PGE_2_ concentrations and MPO activities in the small intestine were similar between the isoflurane-anesthetized normal mice and the urethane-anesthetized sham-operated mice. Furthermore, in this study, no macroscopic and microscopic damages of the small intestine were observed in the sham-operated mice that had been anesthetized with urethane. Accordingly, the damaging effects of urethane on the small intestine and the effects of urethane on the expression of intestinal mediators associated with I/R injury, if any, were considered to be negligible in the adult mice.

In conclusion, TLR2 may mediate I/R injury of the small intestine in adult mice via the induction of inflammatory mediators such as TNF-α and ICAM-1. Therapies that interfere with TLR2-dependent signaling could be effective against adult intestinal I/R injury.
